# Exercise-Induced Autophagy Suppresses Sarcopenia Through Akt/mTOR and Akt/FoxO3a Signal Pathways and AMPK-Mediated Mitochondrial Quality Control

**DOI:** 10.3389/fphys.2020.583478

**Published:** 2020-11-02

**Authors:** Zhengzhong Zeng, Jiling Liang, Liangwen Wu, Hu Zhang, Jun Lv, Ning Chen

**Affiliations:** ^1^Graduate School, Wuhan Sports University, Wuhan, China; ^2^Sports Institute, Chongqing University of Arts and Sciences, Chongqing, China; ^3^Tianjiu Research and Development Center for Exercise Nutrition and Foods, Hubei Key Laboratory of Exercise Training and Monitoring, College of Health Science, Wuhan Sports University, Wuhan, China

**Keywords:** sarcopenia, exercise intervention, autophagy, Akt/mTOR signal pathway, Akt/FoxO3a signal pathway, mitochondrial quality control

## Abstract

Exercise training is one of the most effective interventional strategies for sarcopenia in aged people. Nevertheless, the underlying mechanisms are not well recognized. Increasing studies have reported abnormal regulation of autophagy in aged skeletal muscle. Our current study aims to explore the efficiency of exercise interventions, including treadmill exercise, resistance exercise, alternating exercise with treadmill running and resistance exercise, and voluntary wheel running, on 21-month-old rats with sarcopenia and to detect the underlying mechanisms. Results showed the declined mass of gastrocnemius muscle with deficient autophagy and excessive apoptosis as a result of up-regulated Atrogin-1 and MuRF1, declined Beclin1 level and LC3-II/LC3-I ratio, accumulated p62, increased Bax, and reduced Bcl-2 levels, and also exhibited a defective mitochondrial quality control due to declined PGC-1α, Mfn2, Drp1, and PINK1 levels. However, 12-week exercise interventions suppressed the decline in mass loss of skeletal muscle, accompanied by down-regulated Atrogin-1 and MuRF1, increased Beclin1 level, improved LC3-II/LC3-I ratio, declined p62 level, and reduced Bax and increased Bcl-2 level, as well as enhanced mitochondrial function due to the increased PGC-1α, Mfn2, Drp1, and PINK1 levels. Moreover, exercise interventions also down-regulated the phosphorylation of Akt, mTOR, and FoxO3a, and up-regulated phosphorylated AMPK to regulate the functional status of autophagy and mitochondrial quality control. Therefore, exercise-induced autophagy is beneficial for remedying sarcopenia by modulating Akt/mTOR and Akt/FoxO3a signal pathways and AMPK-mediated mitochondrial quality control, and resistance exercise exhibits the best interventional efficiency.

## Introduction

As the population undergoes rapid aging, the number of elderly patients with aging-related diseases is also increasing. By 2050, the number of individuals over 65 years old will increase to over 1.5 billion (Maiese, 2018). Aging and aging-related diseases have become a public health problem with potential threats. Skeletal muscle is the largest locomotor and metabolic organ in the human body, which accounts for approximately 40% of body weight, and is the basis of various physical activities (Iizuka et al., 2014). Nevertheless, throughout the aging procedure, the structure and function of the neuromuscular system will unavoidably undertake degenerative modifications, which manifests as the mass loss and strength decrease of skeletal muscle, thereby resulting in the reduction of endurance and metabolic capacity, and the accumulation of connective tissue and fat in skeletal muscle (Larsson et al., 2019). Sarcopenia is a chronic aging-related disease characterized by progressive loss of mass and function of skeletal muscle, thereby leading to physical disability, poor quality of life, and even death. The pathogenesis of sarcopenia includes inflammation, apoptosis, disordered protein synthesis and degradation, and dysfunctional mitochondria (Chumlea et al., 2011; Zembron-Lacny et al., 2014). Autophagy is an extremely conserved catabolic procedure in the cytoplasm of eukaryotes, through which abnormal organelles are transported into lysosomes for degradation to perform recycling and renewal of organelles. Therefore, autophagy is necessary for maintaining cellular homeostasis (Bellot et al., 2009) and plays an essential function in metabolism, structure reconstruction, growth, and development (Bento et al., 2016). Previous studies have demonstrated a close correlation between the functional status of autophagy and the atrophy of skeletal muscle (Masiero et al., 2009), so that both excessive autophagy and deficient autophagy can lead to muscular atrophy (Sandri, 2010). Maintaining or optimizing the functional status of autophagy can delay skeletal muscle atrophy and improve skeletal muscle function. Although multiple interventions, such as dietary supplements, calorie limitation, and physical exercise, on alleviating sarcopenia have been widely discussed, exercise intervention may be the appropriate approach (Phu et al., 2015). Exercise can increase skeletal muscle mass and strength and alleviate sarcopenia (Cruz-Jentoft and Landi, 2014; Landi et al., 2014); however, the underlying mechanisms are still not fully clear. At the same time, a multitude of studies have actually revealed that exercise training can significantly promote autophagy in the skeletal muscle of aged animals (Grumati et al., 2011; Hamurcu et al., 2018); in addition, Akt/mTOR and Akt/FoxO3a signal pathways play important regulatory roles for autophagy in skeletal muscle (Mammucari et al., 2008). Mitochondria are not only the energy factories of cells, but also the regulatory centers of cell signal transduction, and the mitochondrial quality control system restricts abnormal accumulation of mitochondria through mitochondrial biogenesis, mitochondrial fission/fusions, or mitophagy, so as to ensure the relative stability of mitochondrial quantity and quality and the performance of normal mitochondrial physiological functions. Mitochondrial quality control in skeletal muscle is closely related to sarcopenia, and dys-regulated mitochondrial quality control leads to sarcopenia in naturally or rapidly aged mice (Joseph et al., 2013; Liu et al., 2020). Previous studies have shown that exercise-induced AMPK can regulate energy metabolism and promote mitochondrial biosynthesis in skeletal muscle, participate in the quality control of skeletal muscle, regulate inflammatory responses, and inhibit muscular atrophy (Brault et al., 2010; Drake et al., 2016; Liu and Chang, 2018). Therefore, we hypothesize that exercise can regulate Akt/mTOR and Akt/FoxO3a signal pathways to induce autophagy and cooperate with AMPK-mediated mitochondrial quality control to alleviate sarcopenia. The atrophy of type II myofibers in skeletal muscle is a prominent feature of sarcopenia (Sayed et al., 2016), so the gastrocnemius muscle was selected as the subject of this study.

## Materials and Methods

### Animal Management

For the experiment, 60 6-month-old male Wistar rats (body weight: 450 ± 50 g; No: 42000600020738) were ordered from the Experimental Animal Center for Hubei Provincial Center for Disease Control and Prevention (Wuhan, China). All animals were raised with standard food and water with free accessibility under a specific pathogen-free (SPF)-grade environment with a room temperature of 22–24°C and a 12 h dark-light cycle. The experimental protocols were approved by the Institutional Animal Care and Use Committee at Wuhan Sports University. Upon reaching 21 months of age, rats were randomly divided into five groups consisting of the normally aged model group (OC), treadmill exercise group (OT), resistance exercise group (OR), alternating exercise with treadmill and resistance exercise group (OM), and voluntary wheel running group (OV), with 10 mice in each group. Another 10 6-month-old Wistar rats were utilized as the young control group (YC). After exercise interventions for 12 successive weeks, all rats fasted for 24 h and were sacrificed by decapitation to gather experimental samples. Subsequently, the harvested gastrocnemius muscle samples were quickly frozen in liquid nitrogen and afterwards stored at −80°C freezer.

### Exercise Protocols

#### Treadmill Exercise Protocol

The rats went through treadmill running with weight bearing at 6:00–8:00 pm 3 days per week (once every other day). In order to adapt to the apparatus and avoid subsequent exercise-induced stress, the rats were placed on the treadmill for 30 min without running on the first day. After adaptive training on the first day, the rats began running at a speed of 4.2 m/min, with a progressive increase to a final speed of 12 m/min at a speed increment of 1 m/min every 30 s, and then maintained this speed for exercise training for 12 weeks with 60 min during each training time.

#### Resistance Exercise Protocol

As mentioned previously, resistance exercise was carried out through ladder-climbing training (Aamann et al., 2019). The ladder was 1 m in height with 2 cm steps and an 85° incline. Loads were also affixed to the rats’ tails for ladder-climbing training at 6:00–8:00 pm 3 days per week (once every other day). At the beginning, the rats were offered a load comparable to 10% of their body weight, and progressively loads were increased at a rate of 10% body weight weekly, finally reaching 80% of body weight. The training consisted of two sets with three repetitions, followed by 1 min rest between each repetition and 2 min rest between each set.

#### Alternating Exercise With Treadmill Running and Resistance Exercise

Treadmill running and ladder-climbing training were alternated at 6:00–8:00 pm 3 days per week (once every other day) based on the above-mentioned protocols.

#### Voluntary Wheel Running Protocol

The rats were provided with a free lifestyle for living in the cage with a rotating running wheel (diameter in 33 cm and width in 10 cm) and rats were able to climb the running wheel freely for voluntary exercise.

### Transmission Electron Microscopic Examination

The transmission electron microscopic assay was executed as illustrated previously (Chen et al., 2020); in brief, 1 mm^3^ of gastrocnemius muscle sample was fixed, rinsed, cut (70 nm), and stained. The sections were subsequently examined and analyzed under a transmission electron microscope (TEM, HT7700, Hitachi, Japan) at the Research Center for Medicine and Structural Biology of Wuhan University.

### Histological Assay

After euthanizing the rats, gastrocnemius muscle samples were harvested and embedded in Tissue-Tek OCT (optimum cutting temperature) compound and stored at −80°C after being snap-frozen in liquid nitrogen. After being cut at 5 μm, the sections were dewaxed, rehydrated, and stained with hematoxylin-eosin (HE). The sample sections from each group were observed under a light microscope (Eclipse-E100, Nikon, Japan) with an imaging system (DS-U3, Nikon, Japan) at 400x zoom. The cross-sectional areas (CSA) of 150 skeletal muscle fibers were gauged using ImageJ software.

### Western Blot Analysis

As detailed formerly (Lee et al., 2019), total proteins (40 μg) underwent 10 or 12% SDS-polyacrylamide gel (SDS-PAGE) and were electroblotted onto polyvinylidene fluoride (PVDF) membranes. Then, the target protein was blocked and probed at 4°C for 12 h with primary antibodies against PGC-1α, Atrogin-1, or MuRF1 (rabbit polyclonal, 1:1000; Abcam, Cambridge, MA, United States), Beclin1, p62 (SQSTM1/Sequestome 1), LC3 (microtubule-associated protein-1 light chain 3, LC3), Bcl-2, Bax, AMPK, Mfn2, Drp1, PINK1, p-AMPK^Thr172^, Akt, p-Akt^Ser473^, mTOR, p-mTOR^Ser2448^, FoxO3a, p-FoxO3a^Ser253^ (rabbit polyclonal, 1:1000; Cell Signaling Technology, Danvers, MA, United States), and GAPDH (rabbit polyclonal, 1:5000; Cell Signaling Technology, Danvers, MA, United States). After being washed three times for 15 min in 0.1% TBS-T buffer, the membrane was incubated with goat horseradish peroxidase (HRP)-conjugated goat anti-rabbit IgG (1:10000, Cell Signaling Technology, Danvers, MA, United States) at room temperature for 1 h, and the target protein was detected by enhanced chemiluminescence (ECL) reagent imaged by ultra-sensitive fluorescence/chemiluminescence imaging system ChemiScope6300 (CLiNX Science Instruments, Shanghai, China).

### Statistical Analysis

All data were expressed as mean ± standard deviation (M ± SD). The difference among all experimental groups was analyzed using one-way ANOVA through IBM SPSS Statistics 20 (IBM, New York, NY, United States). The significant difference was considered at *p* < 0.05.

## Results

### Exercise Interventions Rescued the Atrophy of Skeletal Muscle in Aged Rats

It is well recognized that aging is related to a slowly progressive decline of mass and function in skeletal muscle. Consequently, the reduction of skeletal muscle mass is dependable evidence for the aging process. To detect the mass loss of skeletal muscle in aged rats, we examined gastrocnemius muscle weight-body weight ratio (GMW/BW) as the sarcopenia index (SI) for indicating an indirect indicator of skeletal muscle atrophy (Edstrom et al., 2007; Kashani et al., 2017). As shown in Table 1, the SI value of the rats from the OC group showed a considerable decline when compared to that in the YC group. On the other hand, exercise interventions, especially resistance exercise, can significantly increase the SI values of aged rats and rescue aging-induced atrophy of skeletal muscle.

**Table 1:** Exercise interventions rescued the reduced GMW/BW ratio in aged rats (*n* = 10).

Groups	Body weight (g)	Gastrocnemius muscle (g)	GMW/BW ratio (×100)
YC	490.2 ± 35.866	6.373 ± 0.644	1.302 ± 0.112
OC	867.9 ± 35.967	6.766 ± 0.88	0.783 ± 0.124^***^
OT	700.6 ± 37.542	6.508 ± 0.697	0.93 ± 0.105^##^
OR	757.5 ± 27.257	8.298 ± 1.303	1.095 ± 0.159^###^
OM	794.2 ± 35.683	7.283 ± 0.675	0.917 ± 0.081^#^
OV	672.5 ± 24.277	6.634 ± 0.797	0.986 ± 0.107^###^

The ratio is calculated from gastrocnemius muscle weight (GMW)/body weight (BW). All data are presented as mean ± standard deviation (M ± SD) from 10 rats in each group (n = 10).

*** *p* < 0.001 vs. YC group

# *p* < 0.05

## *p* < 0.01

### *p* < 0.001 vs. OC group.

**Figure 1:**
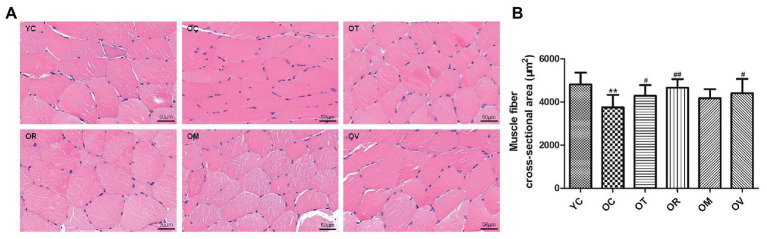
Exrcise interventions increased the cross-sectional area (CSA) of skeletal muscle fibers in aged rats. **(A)** Morphological changes of skeletal muscle were observed with HE staining (scale bar, 50 μm). **(B)** The CSA of skeletal muscle fibers in aged rats was measured from the selected 150 gastrocnemius muscle fibers. ^**^p < 0.01 vs. YC group; ^#^*p* < 0.05 and ^##^*p* < 0.01 vs. OC group. All data are presented as mean ± standard deviation (M ± SD) from the selected 150 gastrocnemius muscle fibers in each group (n = 150).

Under normal circumstances, myofilaments showed a regular arrangement, following the nucleus situated at the side of skeletal muscle fibers; however, irregular shapes and nucleus aggregation were observed in the skeletal muscle of the rats from the OC group. After exercise interventions, the irregular structure of skeletal muscle fibers was clearly improved (Figure 1A). Meanwhile, compared to the YC group, the CSA of skeletal muscle fibers were extraordinarily reduced in aged rats. However, a significant reversal was detected in the OT, OR, and OV groups (Figure 1B), suggesting that the aging-induced atrophy of skeletal muscle could be alleviated by regular exercise interventions.

**Figure 2:**
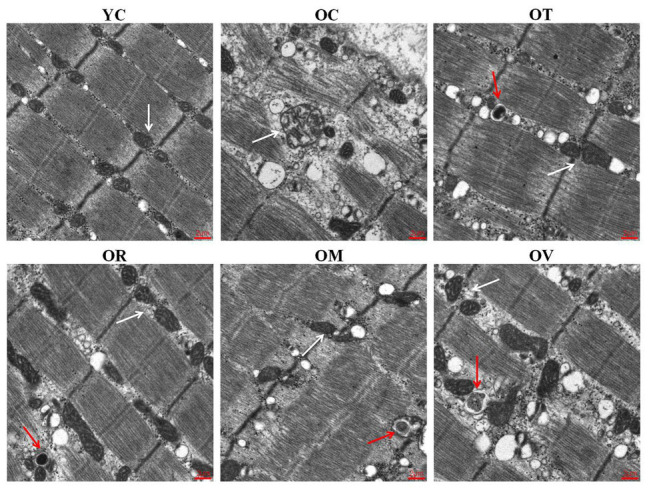
Exercise could repair the damaged structure of skeletal muscle fibers in aged rats examined by transmission electron microscope (scale bar, 2 μm). White arrows labeled are mitochondria, and red arrows labeled are autophagosomes in gastrocnemius muscle.

The aging of skeletal muscle is characterized by damaged or swollen mitochondria, disordered arrangement of myofilaments, or irregular fiber structure. Compared to the YC group, skeletal muscle from the OC group revealed a disordered arrangement of myofilaments and swollen and vacuous mitochondria. However, the ordered arrangement of myofilaments, normal mitochondria, and autophagosomes were observed in the skeletal muscle of rats from the OT, OR, OM, and OV groups (Figure 2), suggesting that exercise interventions can alleviate the aging process of skeletal muscle.

### Exercise Suppressed E3 Ubiquitin Ligase in Skeletal Muscle of Aged Rats

**Figure 3:**
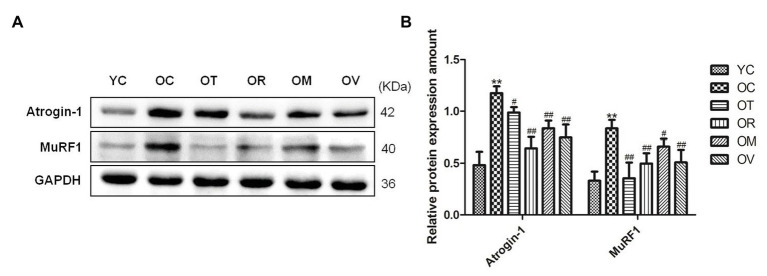
Western blots of E3 ubiquitin ligase proteins. **(A)** Atrogin-1 and MuRF1 were subjected to Western blot analysis using corresponding antibodies. **(B)** Quantitative analysis is corrected for loading with GAPDH. ^**^p < 0.01 vs. YC group; ^#^*p* < 0.05 and ^##^*p* < 0.01 vs. OC group. All data are presented as mean ± standard deviation (M ± SD) from independent experiments performed in triplicate (n = 3) in each group.

E3 ubiquitin ligases including Atrogin-1 (atrophy gene-1) and MuRF1 (muscle ring finger protein 1) play a crucial role in the atrophy of skeletal muscle (Bodine et al., 2001). As shown in Figures 3A,B, higher expression levels of Atrogin-1 and MuRF1 in gastrocnemius muscles of OC rats were observed when compared to YC rats; however, exercise interventions significantly down-regulated Atrogin-1 and MuRF1 expression, indicating that exercise interventions may have the function to suppress protein ubiquitination in the skeletal muscle of aged rats and alleviate the atrophy of skeletal muscle.

### Exercise Induced Autophagy and Inhibited Excessive Apoptosis in the Skeletal Muscle of Aged Rats

**Figure 4:**
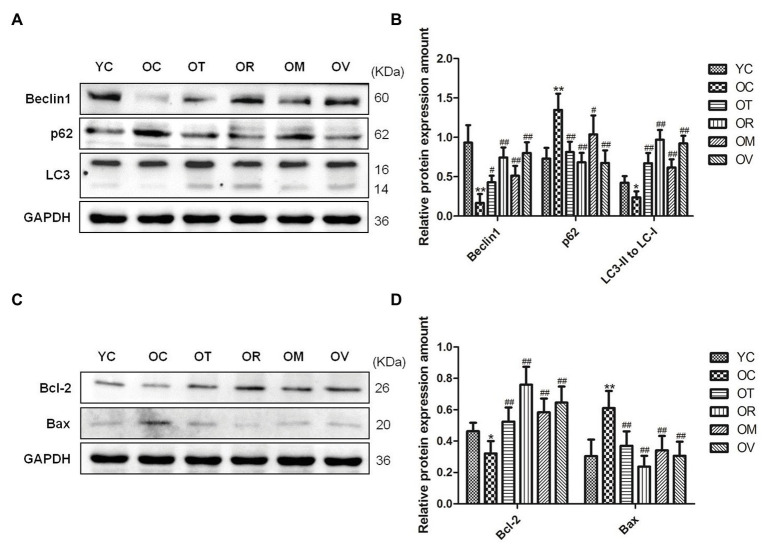
Western blots of autophagy and apoptosis-related proteins. **(A,C)** Beclin1, p62, LC3, Bcl-2, and Bax were subjected to Western blot analysis using corresponding antibodies. **(B,D)** Quantitative analysis is corrected for loading with GAPDH. ^*^p < 0.05 and ^**^p < 0.01 vs. YC group; ^#^*p* < 0.05 and ^##^*p* < 0.01 vs. OC group. All data are presented as mean ± standard deviation (M ± SD) from independent experiments performed in triplicate (n = 3) in each group.

Both autophagy and apoptosis are highly correlated with biological procedures, and play an essential function in differentiation, development, and homeostasis in an interdependent or interrelated manner. In the present study, lower Beclin1 expression and LC3-II/LC3-I ratio and higher p62 expression in the skeletal muscle of the rats from the OC group were measured. In contrast, exercise interventions, especially resistance exercise, significantly up-regulated Beclin1 expression, increased LC3-II/LC3-I ratio, and down-regulated p62 expression (Figures 4A,B), suggesting lower autophagic flux, and rescued the abnormal status of autophagy in the skeletal muscle of the aged rats upon exercise interventions. To determine the anti-apoptotic activity, the protein expression of Bcl-2 and Bax was evaluated. Lower Bcl-2 levels and higher Bax levels were measured in the skeletal muscle of rats from the OC group; nevertheless, exercise interventions, especially resistance exercise, could result in the increased expression of Bcl-2 and decreased expression of Bax (Figures 4C,D), indicating that the protection mechanism in reaction to exercise is to sufficiently inhibit excessive apoptosis in the skeletal muscle of aged rats.

### Exercise Regulated Akt/mTOR and Akt/FoxO3a Signal Pathways in Skeletal Muscle of Aged Rats

**Figure 5:**
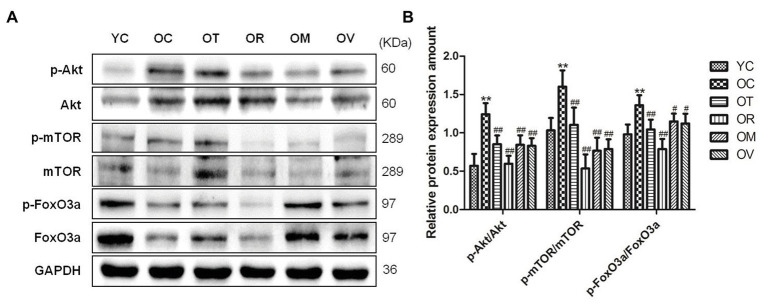
Western blots of Akt/mTOR and Akt/FoxO3a signal pathways related proteins. **(A)** The p-Akt^Ser473^, Akt, p-mTOR^Ser2448^, mTOR, p-FoxO3a^Ser253^, and FoxO3a were subjected to Western blot analysis using corresponding antibodies. **(B)** Quantitative analysis is corrected for loading with GAPDH. ^**^p < 0.01 vs. YC group; ^#^*p* < 0.05 and ^##^*p* < 0.01 vs. OC group. All data are presented as mean ± standard deviation (M ± SD) from independent experiments performed in triplicate (n = 3) in each group.

Autophagy can be enhanced by modulating Akt/mTOR and Akt/FoxO3a signal pathways (Jung et al., 2010). The p-Akt^Ser473^/Akt, p-mTOR^Ser2448^/mTOR, and p-FoxO3a^Ser253^/FoxO3a ratios in the skeletal muscle of the OC group were detected to reveal a significant increase, whereas exercise interventions, especially resistance exercise, could decrease the ratios as above (Figures 5A,B), indicating exercise interventions can recover the deficiency of autophagy in the skeletal muscle of aged rats by modulating Akt/mTOR and Akt/FoxO3a signal pathways.

### Exercise Induced AMPK to Regulate Mitochondrial Quality Control in the Skeletal Muscle of Aged Rats

**Figure 6:**
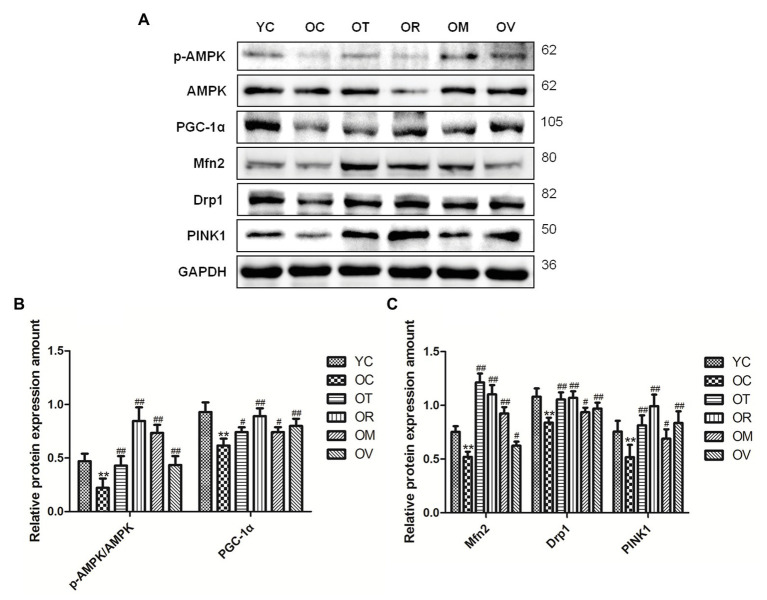
Western blots of mitochondrial quality control related proteins. **(A)** The p-AMPK^Thr172^, AMPK, PGC-1α, Mfn2, Drp1, and PINK1 were subjected to Western blot analysis using corresponding antibodies. **(B,C)** Quantitative analysis is corrected for loading with GAPDH. ^**^p < 0.01 vs. YC group; ^#^*p* < 0.05 and ^##^*p* < 0.01 vs. OC group. All data are presented as mean ± standard deviation (M ± SD) from independent experiments performed in triplicate (n = 3) in each group.

As a highly conserved serine/threonine-protein kinase, AMPK plays an essential role in mitochondrial quality control. As shown in Figures 6A–C, lower p-AMPK^Thr172^/AMPK ratio, PGC-1α, Mfn2, Drp1, and PINK1 expression were measured in the skeletal muscle of rats from the OC group, whereas exercise interventions, especially resistance exercise, could up-regulate their expression in the skeletal muscle of aged rats, suggesting that exercise can activate AMPK to regulate mitochondrial quality control in the skeletal muscle of aged rats.

## Discussion

A progressive reduction of skeletal muscle mass and strength during the aging process is recognized as sarcopenia. As the most intuitive indicator to sarcopenia, the CSA of skeletal muscle fibers exhibits a continuous decrease with the aging process (Klitgaard et al., 1990; Verdijk et al., 2007). Previous studies have reported that both resistance exercise and treadmill exercise can significantly increase the CSA of gastrocnemius muscle in aged Wistar and SD rats (Liao et al., 2017; de Sousa Neto et al., 2020). Because type II muscle fiber is the most prominent fiber type in the lateral gastrocnemius, it thereby exhibits greater gains when compared with the medial gastrocnemius for muscle hypertrophy caused by resistance exercise in young men (Schoenfeld et al., 2020). In our study, exercise interventions, especially resistance exercise, significantly increased the CSA of the gastrocnemius muscle in aged rats, which may be associated with the hypertrophy of specific type II muscle fibers. In addition, whether autophagy and AMPK signaling can be down-regulated and its potential integration with sarcopenia in the gastrocnemius muscle of normally aged rats were also explored in the present study. Meanwhile, whether treadmill exercise, resistance exercise, alternating exercise with treadmill running and resistance exercise, or voluntary wheel running can recover sarcopenia by regulating autophagy and AMPK signaling was investigated. Comparable with previous research (Fan et al., 2017), a significant down-regulation of autophagy and phosphorylation of AMPK^Thr172^ in D-gal-induced aged rats was observed, along with increased apoptosis levels and skeletal muscle mass loss or atrophy. As assumed, four types of exercise interventions can rescue sarcopenia by recovering autophagy and activating the AMPK signal pathway.

In mammalian cells, autophagy is mainly regulated by autophagy-related proteins. Beclin1 is the first autophagy-related gene found in mammals. As one of the most important molecules in the formation of autophagosomes, Beclin1 is a key target for regulating the autophagy activity of cells (Tsuchihara et al., 2009; He and Levine, 2010). During autophagy, autophagy-related proteins 8 (Atg8) plays an essential role in the formation of autophagosomes in yeasts and plants. The animal homolog of the Atg8 protein is LC3; the formation of the phagophore causes the transformation of LC3-I into LC3-II through lipidation. Therefore, as a critical marker of autophagy induction, LC3-II/LC3-I ratio can estimate the functional status of autophagy (Klionsky et al., 2011; He et al., 2015). p62 is a multifunctional scaffold protein consisting of numerous domains, can bind ubiquitinated proteins, and also target them for degradation by proteasomes. In addition, p62 can straightly bind LC3, which might deliver ubiquitinated proteins for degradation by autophagy (Pankiv et al., 2007). Previous studies have actually shown decreased levels of autophagy in the skeletal muscle of elderly animals (Cuervo et al., 2005; Bergamini, 2006; Salminen and Kaarniranta, 2009), while exercise intervention can activate autophagy of aged skeletal muscle (White et al., 2016). Our results have additionally confirmed that four kinds of exercise interventions, especially resistance exercise, can rescue autophagy levels of aged rats and improve the autophagic flux of skeletal muscle cells as a result of up-regulated Beclin1 and down-regulated p62, along with increased LC3-II/LC3-I ratio (Figures 4A,B). Bcl-2 and Bax are a pair of important proteins for regulating autophagy and apoptosis, and both come from the Bcl-2 genetic family members. Bcl-2 is an apoptosis suppressor that can inhibit apoptosis by inhibiting glutathione exhalation, cytochrome C release, and the activation of apoptotic protease (Zhao et al., 2015). Bax is a homolog of Bcl-2 that promotes apoptosis by destroying the integrity of mitochondria, and also attenuates the anti-apoptotic effect of Bcl-2 by binding to Bcl-2 (Tan et al., 2005). The balance between Bax and Bcl-2 proteins can determine the fate of cells. Bcl-2 can not only inhibit apoptosis, but also regulate autophagy by binding to Beclin1. Therefore, the relationship between Bcl-2 and Beclin1 is very important to regulate autophagy and apoptosis (Xu and Qin, 2019). Studies have shown that regular exercise increases the expression of Bcl-2 protein, and inhibits the expression of Bax protein, thereby significantly reducing DNA fragmentation caused by aging, and alleviating the atrophy of skeletal muscle (Song et al., 2006). Consistent with the above findings, our results also indicate that four kinds of exercise interventions, especially resistance exercise, can inhibit excessive apoptosis with up-regulated Bcl-2 and down-regulated Bax in the skeletal muscle of aged rats (Figures 4C,D).

mTOR is a serine/threonine-protein kinase that can negatively regulate autophagy (Proud, 2007). mTOR inhibition can also activate ULK1, which phosphorylates Beclin1^S14^, and therefore enhances the activity of the Atg14L-VPS34 complexes and autophagy induction (Russell et al., 2013). FoxO comes from the large family of forkhead transcription factors that are extensively distributed in all kinds of eukaryotic organisms, including FoxO1, FoxO3a, and FoxO4, in skeletal muscle (Birkenkamp and Coffer, 2003; Zheng et al., 2019). FoxO3a is needed for autophagy induction by regulating the transcription of autophagy-related genes such as LC3 and BNIP3 (Mammucari et al., 2007). Akt, also known as protein kinase B (PKB), comes from the serine/threonine-protein kinase family, and negatively regulates autophagy by activating mTOR and phosphorylating FoxO3a (Jung et al., 2010). Akt/FoxO3a is considered one of the major signal pathways that can regulate autophagy in skeletal muscle by inducing LC3 (Mammucari et al., 2008), and studies have found that chronic resistance exercise could up-regulate Beclin1 by suppressing the phosphorylation of AKT^Ser473^ and mTOR^Ser2448^ in the skeletal muscle of aged rats (Luo et al., 2013). In this study, the increased phosphorylation of Akt^Ser473^, mTOR^Ser2448^, and FoxO3a^Ser253^ in the skeletal muscle of aged rats is also significantly reversed from exercise groups, especially the OR group (Figures 5A,B). It suggests that exercise interventions, especially resistance exercise, might induce autophagy in aged skeletal muscle by moderating Akt/mTOR and Akt/FoxO3a signal pathways to promote the expression of autophagy-related proteins, such as Beclin1 and LC3. It is worth noting that mTOR^Ser2448^ is not only the target of upstream signal AKT kinase, but also is a target of p70S6K, as part of a feedback mechanism. In addition, mTORC1 is also a target of its downstream p70S6K, so mTOR^Ser2448^ phosphorylation may be the parallel change in mTORC1 activity under certain circumstances. Furthermore, the latest studies have shown that p70S6K^Thr389^ is considered a direct and reliable target to estimate mTORC1 activity (Figueiredo et al., 2020). Therefore, it is highly necessary to conduct experimental validation for this parameter in future studies.

In general, mitochondria are the most important organelles for energy production, free radical metabolism, and programmed cell death. Mitochondrial dysfunction can lead to the metabolic disorders of reactive oxygen species (ROS), thus resulting in the activation of DNA damage response and cell aging. Mitochondrial quality control ensures the relative stability of the quantity and quality of mitochondria, maintains mitochondrial homeostasis through regulating mitochondrial biosynthesis, mitochondrial dynamic balance and mitophagy, and alleviates cell aging; in contrast, dysregulated mitochondrial quality control can contribute to sarcopenia (Liu et al., 2020).

Mitochondrial biogenesis is a complicated procedure that promotes a development in mitochondrial content to fulfill cellular energy as needed. PGC-1 is the master regulator of mitochondrial biogenesis. Various factors leading to the increase of AMP/ATP ratio, such as ischemia, hypoxia, exercise, and hunger, can activate AMPK or stimulate its expression (Jensen et al., 2009), which then promotes mitochondrial biogenesis by up-regulating PGC-1α (Herzig and Shaw, 2018). In this research, the phosphorylation of AMPK^Thr172^ and the expression of PGC-1α are increased after exercise interventions in aged skeletal muscle, thus affirming that exercise interventions, especially resistance exercise, can activate the AMPK/PGC-1α signal pathway and promote mitochondrial synthesis in the skeletal muscle of aged rats (Figures 6A,B).

Mitochondrial dynamics, including fusion and fission, have been acknowledged as essential events in mitochondrial metabolism. Several studies on the molecular mechanism of exercise-mediated mitochondrial fusion report variant results, ranging from a significant decrease, to a little bit of change, to a noticeable increase (Bori et al., 2012; Kitaoka et al., 2015, 2016). These inconsistencies may arise from the variety of exercise modes and intensities. In our current study, we have measured the mitochondrial fusion-related protein Mfn2 to reveal a significant increase in the skeletal muscle of aged rats upon exercise interventions. Additionally, compared to the stress response of mitochondrial fusion to exercise, studies have found that the expression of genes related with mitochondrial fission is up-regulated significantly after a single bout or long-term exercise training (Ding et al., 2010; Bori et al., 2012). Moreover, the expression level of mitochondrial fission protein reveals a decrease in aged mice (Joseph et al., 2013). Drp1 is a key molecule involved in mitochondrial fission and plays an important role in maintaining skeletal muscle mass (Favaro et al., 2019). Previous studies have revealed that activated AMPK can phosphorylate mitochondrial fission factor, recruit Drp1 to situate in the mitochondrial external membrane, and to trigger mitochondrial fission, thereby inducting mitophagy (Zhang and Lin, 2016). Stress factors, such as exercise and energy restriction, can also activate AMPK and promote mitophagy to selectively degrade mitochondria and control the number of mitochondria (Sancak et al., 2010), which is important for conserving the regular phenotypes and functions of cells. PINK1/Parkin is the most classic mitophagy signal pathway. Under conditions of stress, injury, or aging, PINK1 assembles on the external membrane of dysfunctional mitochondria, where it activates and also recruits E3 ubiquitin ligase Parkin to trigger mitophagy by ubiquitinating outer mitochondrial membrane proteins. In the current study, the phosphorylation of AMPK^Thr172^ and the expression of Drp1 and PINK1 proteins are increased after exercise interventions in aged skeletal muscle, suggesting that exercise interventions, especially resistance exercise, can promote AMPK-mediated mitophagy (Figures 6A,C).

The ubiquitin proteasome pathway applies to the whole cell and involves numerous substrates and reaction processes *in vivo*, including substrate protein ubiquitination and degradation of ubiquitinated proteins. The E3 ubiquitin ligases, such as Atrogin-1 and MuRF1, are essential in protein ubiquitination. They selectively identify substrate proteins and promote ubiquitin transfer to substrate proteins; through this, then ubiquitinated protein substrate is finally recognized and degraded by 26S proteasome in skeletal muscle. Previous studies have found that E3 ubiquitin ligase, including Atrogin-1 and MuRF1, are closely related to skeletal muscle atrophy, and are significantly up-regulated in a variety of skeletal muscle atrophy models (Li et al., 2003; Foletta et al., 2011; Wang et al., 2011). In contrast, exercise training can decrease the expression of Atrogin-1 and MuRF1 in aged skeletal muscle (Ribeiro et al., 2017). In addition, Atrogin-1, MuRF1, and protein ubiquitination show similar changes in various aging tissues (Ziaaldini et al., 2015; Kou et al., 2017), and exercise training can reverse the aging-associated activation of catabolic signaling to fight sarcopenia (Ziaaldini et al., 2015). In this study, exercise interventions, especially resistance exercise, significantly decreased the expression of Atrogin-1 and MuRF1 proteins in the skeletal muscle of aged rats (Figures 3A,B), which may subsequently inhibit the process of protein ubiquitination. The regulation of Atrogin-1 and MuRF1, including FoxO3a and NF-κB signal pathways, has a cumulative effect on skeletal muscle atrophy (Reed et al., 2011), of which NF-κB is one of the most important transcription factors to regulate Atrogin-1 and MuRF1 involved in aged skeletal muscle atrophy (Bar-Shai et al., 2008; Bodine and Baehr, 2014). Furthermore, the overexpression of PGC-1α or activation of the AMPK/PGC-1α signal pathway can inhibit the transcriptional activity of NF-κB, and also decline Atrogin-1 and MuRF1 expression, thereby postponing muscular atrophy (Brault et al., 2010; Kim and Lee, 2017; Liu and Chang, 2018). Therefore, exercise training can reduce the expression of Atrogin-1 and MuRF1 proteins, which might be partly due to the activation of the AMPK/PGC-1α signal pathway in aged rats.

**Figure 7:**
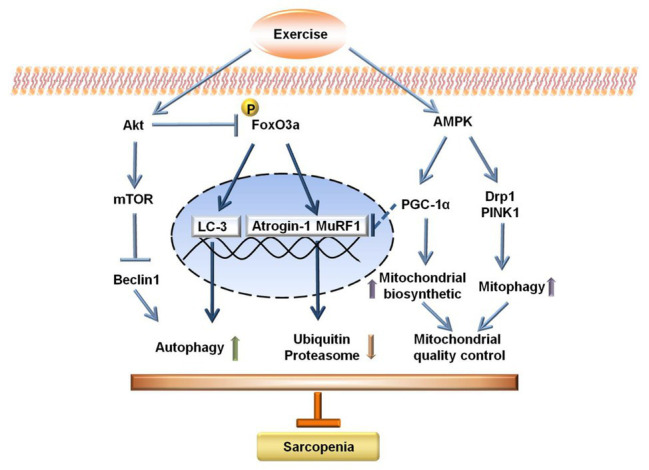
Schematic diagram for the potential mechanisms of sarcopenia upon exercise interventions.

In summary, exercise interventions, especially resistance exercise, can induce autophagy, maintain mitochondrial quality control, and suppress E3 ubiquitin ligase in aged skeletal muscle, which may provide the exercise-induced advantageous effect on alleviating sarcopenia. Mechanistically, the modulation of the Akt/mTOR, Akt/FOXO3a, and AMPK signal pathways are implicated in the mitigation of sarcopenia upon exercise interventions (Figure 7).

## Data Availability Statement

All datasets presented in this study are included in the article/supplementary material.

## Ethics Statement

The animal study was reviewed and approved by Institutional Animal Care and Use Committee at Wuhan Sports University.

## Author Contributions

ZZ and NC conceived and designed the project. ZZ, JiL, LW, HZ, and JuL performed experiments. ZZ, JiL, HZ, and NC analyzed data and prepared figures. ZZ, JiL, and NC drafted the manuscript. NC edited and revised the manuscript. ZZ, JiL, LW, HZ, JuL, and NC approved the final version of the manuscript. All authors contributed to the article and approved the submitted version.

### Conflict of Interest

The authors declare that the research was conducted in the absence of any commercial or financial relationships that could be construed as a potential conflict of interest.
